# B and N isolate-doped graphitic carbon nanosheets from nitrogen-containing ion-exchanged resins for enhanced oxygen reduction

**DOI:** 10.1038/srep05184

**Published:** 2014-06-05

**Authors:** Lei Wang, Peng Yu, Lu Zhao, Chungui Tian, Dongdong Zhao, Wei Zhou, Jie Yin, Ruihong Wang, Honggang Fu

**Affiliations:** 1 Key Laboratory of Functional Inorganic Material Chemistry, Ministry of Education of the People's Republic of China, Heilongjiang University, Harbin, 150080, P. R. China

## Abstract

B,N-codoped carbon nanostructures (BNCS) can serve as alternative low-cost metal-free electrocatalysts for oxygen reduction reactions (ORR). However, the compensation effect between the p- (B atoms) and n-type (N atoms) dopants would make the covalent boron-nitride (BN) easily formed during the synthesis of BNCS, leading to a unsatisfactory ORR activity. Therefore, it has been challenging to develop facile and rapid synthetic strategies for highly active BNCS without forming the direct covalent BN. Here, a facile method is developed to prepare B and N isolate-doped graphitic nanosheets (BNGS) by using iron species for saving N element and simultaneous doping the B element from nitrogen-containing ion-exchanged resins (NR). The resulting BNGS exhibits much more onset potential (*E*_onset_) compared with the B-doped graphitic carbon nanosheets (BGS), N-doped graphitic carbon nanosheets (NGS), as well as B,N-codoped disorder carbon (BNC). Moreover, the BNGS shows well methanol tolerance propery and excellent stability (a minimal loss of activity after 5,000 potential cycles) compared to that of commercial Pt/C catalyst. The goog performance for BNGS towards ORR is attributed to the synergistic effect between B and N, and the well electrons transport property of graphitic carbon in BNGS.

The cathodic oxygen reduction reaction (ORR) is the crucial process in fuel cells (FCs) and metal-air batteries^1^,^2^,^3^,^4^,^5^,^6^. Pt-based electrocatalysts have the advantages of relatively low overpotential, high current density and four-electron pathway towards ORR^7^,^8^,^9^. However, they still suffer from serious intermediate tolerance, anode crossover, sluggish kinetics and poor stability. These factors, together with the high cost of Pt and its limited natural reserved, have hindered the large-scale application of FCs and metal-air batteries in various areas. Therefore, extensive efforts have been directed to explore alternative low-cost and high-performance ORR electrocatalysts^10^,^11^,^12^,^13^. In this respect, carbon-based metal-free ORR electrocatalysts have generated a great deal of interest owing to their low-cost, high electrocatalytic activity, well selectivity, and excellent durability^14^,^15^,^16^.

Although the abundant free-flowing π electrons in the sp^2^ carbon materials could make them as potential catalysts for reactions needing electrons of ORR, these π electrons are too inert to be used directly in ORR^17^. Therefore, heteroatom doping of carbon materials is an emerging field^18^,^19^,^20^,^21^,^22^,^23^,^24^. To date, it has been revealed that the carbon π electrons in N-doped electron-rich carbon nanostructures (CNS) can be activated by conjugating with the lone-pair electrons from N dopants, resulting in O_2_ molecules could be reduced on the positively charged C atoms neighboring N^25^,^26^. On the other hand, for B-doped electron-deficient CNS, the vacant 2p_z_ orbital of B conjugates with the carbon π system to extract the electrons, leading to these electrons become greatly active due to the low electronegativity of B. Thus, O_2_ molecules are reduced on the positively charged B sites^27^. Based on the synergistic effect, CNS co-doping with B and N is an effective route to further optimize the carbon-based metal-free ORR electrocatalysts.

Recently, there have been a few studies about B,N-codoped CNS as ORR electrocatalysts, and ORR activities is irregular with the ratios and contents of B/N^28^,^29^,^30^,^31^. Chemical vapor deposition (CVD) is the most common synthetic method for B,N-codoped CNS, which suffers from the complicated process and the resultant products with the uncontrollable dopants-level^32^,^33^,^34^,^35^,^36^. Soon afterwards, a facile one-step route was developed for synthesis of B,N-codoped CNS, such as coannealing the graphite oxide (GO) in the present of boric acid and ammonia^37^. However, the compensation effect between the p- (B atoms) and n-type (N atoms) dopants make the covalent boron-nitride (BN) easily form during the synthesis, which could significantly affect the catalytic performance. Hence, it is necessary to develop a effective method for synthesis of B,N-codoped CNS without BN in it. Recently, it has been reported that two-step method is an effective strategy for synthesis of B,N-codoped CNS, which could avoid the direct combination between B and N atoms^38^. In this process, the GO was annealed in ammonia ambient to form N-doped graphene firstly, then the product was annealed in the present of boric acid (HBO_3_) to synthesize B,N-codoped graphene. Although the two-step method could prevent the combination of B and N, leading to the synthetic B,N-codoped graphene exhibits good performance towards ORR, the high-cost of the material derived from graphite oxide (GO) and the complicate synthesized process would limit the realization in practical application. It inspired us to adopt the nitrogen-containing carbonaceous material as the raw material, after introducing the B resource, followed by a carbonized process to prepare B,N-codoped CNS.

In our previous studies, we had demonstrated that the low-cost ion-exchanged resins could be used as a kind of effective carbon resource for synthesis of functional carbon materials by introducing various guest ions into the backbone of resins via a simple ion-exchanged route^39^,^40^,^41^. Despite the tremendous progress in B- and N-codoped CNS for the ORR, to our best knowledge, there have been no reports of the B and N isolate-doped graphitic carbon nanosheets (BNGS) with controllable content of B and N dopants from ion-exchanged route. Herein, we discuss a facile strategy for synthesis of BNGS via a simple ion-exchanged route by using the nitrogen-containing resins (NR) as the carbon and nitrogen resources. In order to avoid the formation of covalent BN, the [Fe(CN)_6_]^3−^ ions were firstly introduced into the skeleton of NR to form NR-[Fe(CN)_6_]^3−^, subsequently, the B precursor of BO^3−^ ions were introduced to prepare NR-[Fe(CN)_6_]^3−^-BO^3−^ precursor. After carbonization under argon ambient and removal of iron species with hydrochloric acid, the BNGS without the covalent BN could be obtained. During the synthetic process, the iron species not only play as the graphitic catalyst but also play as the effect for saving N. More important, the iron species play the role of separating the B and N. Notably, the B and N content in the final products could be controllable by tuning the concentrations of BO^3−^ in the original ion-exchanged solution and the carbonized temperature. The 2D nanosheets with graphitic nanostructures could be favorable for the electrons transport, meanwhile the existence of B and N could play the synergistic effect, resulting in the good performance of BNGS towards ORR. The present work provides a novel way to synthesize BNGS with controllable dopants-level from a facile and economical route as high activities electrocatalysts, which could push forward the development of fuel cells and metal-air cells.

## Results and discussion

**Figure 1:**
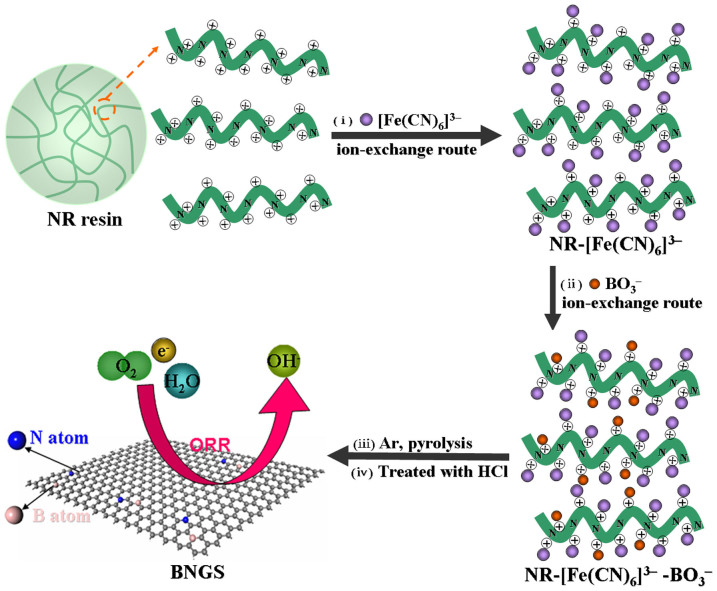
The synthetic processes of BNGS.

The BNGS was synthesized by an ion-exchanged route as illustrated in Figure 1. NR resins was used as the carbon and N resource. Firstly, the [Fe(CN)_6_]^3−^ ions were introduced into the backbone of NR via a ion-exchanged route to form NR-[Fe(CN)_6_]^3−^ composite. Subsequently, the BO^3−^ ions were exchanged into NR to prepare NR-[Fe(CN)_6_]^3−^-BO^3−^ precursor. After carbonization under argon atmosphere and removal of iron catalyst with hydrochloric acid, the BNGS sample was synthesized. In the synthesis, the N atoms were fixed in the backbone of NR, which also could avoid the directly combine between B and N atoms in the BNGS during the carbonized process.

**Figure 2:**
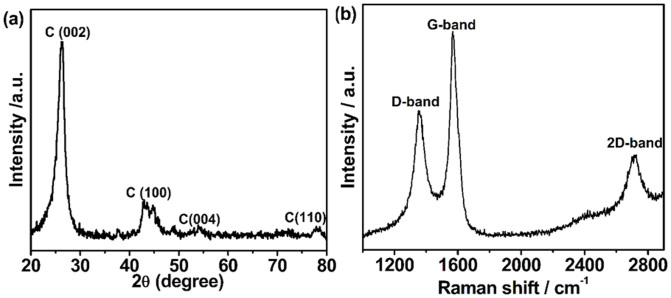
(a) XRD images and (b) Raman spectrum of BNGS-2-900 sample.

Typical X-ray diffraction (XRD) pattern of BNGS derived from 0.02 M HBO_3_ with a carbonized temperature of 900°C (denoted as BNGS-2-900) is shown in Figure 2a. The diffraction peaks at 26.2°, 42.2°, 54.0° and 77.2° are the characteristics of the graphite (002), (100), (004) and (110) planes, respectively, implying the formation of graphitic carbon^42^. Raman is a powerful spectroscopic technique for characterizing carbon materials. As shown in Figure 2b, the G-band at 1568 cm^−1^ is attributed to one of the two E_2g_ modes of the stretching vibrations in the sp^2^ domains of perfect graphite^43^. The D-band at 1354 cm^−1^ is usually assigned to the disorder and imperfection of the carbon crystallites. The second-order D peak (2D-band) at 2712 cm^−1^ is the typical symbol of graphitic carbon. The Raman spectrum in Figure 1b shows narrow, strong and not overlapped D and G bands, and the intense 2D-band, which are the symbols of the graphitic carbon. Generally, the intensity ratio between the G-band and the D-band (*I*_G_/*I*_D_) is related to the crystallinity of carbon materials. The *I*_G_/*I*_D_ of BNGS-2-900 is about 1.51, indicating the formation of graphitic carbon, which is consistent with the XRD results.

**Figure 3:**
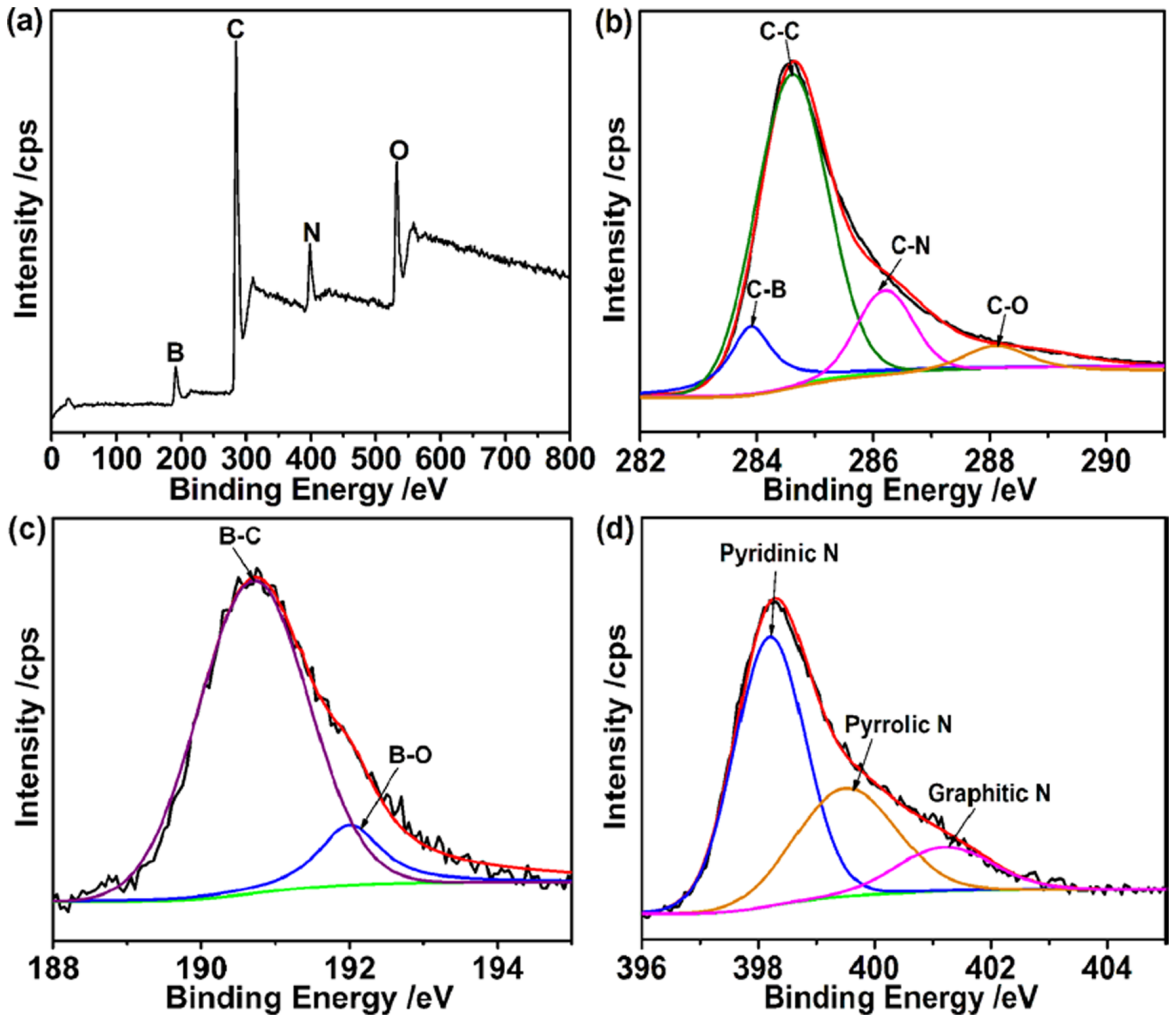
XPS spectrum of BNGS-2-900 sample: (a) survey XPS spectrum and High-resolution XPS spectrum of (b) C1s, (c) B1s and (d) N1s.

X-ray photoelectron spectroscopy (XPS) is a useful spectroscopic technique for measuring the elemental composition, empirical formula, chemical state and electronic state of the elements existed in a material. As shown in Figure 3a, a survey scan indicated that the BNGS-2-900 is composed of C, O, N, B elements. The predominant asymmetric C1s peak shown in Figure 3b implies the existence of C-N or C-B bonds in the graphitic network. The four deconvoluted peaks in the high-resolution C1s spectrum at 283.4, 284.7, 286.2 and 288.3 eV could be attributed to C-B, C-C, C-N and C-O bonds, respectively. On the basis of the XPS analyses, the B and N contents of BNGS-2-900 sample are about 4.40 and 5.12 at.%, respectively. The high-resolution B1s peak in Figure 3c could be divided into two peaks at 190.6 and 192.0 eV, respectively, arising from the B-C (ca. 3.87 at.%) and B-O (ca. 0.53 at.%) bonds^35^,^44^. Obviously, the peak of B-C is major in the BNGS-2-900 sample, indicating that the B atoms mainly attach to C atoms in the network of graphitic carbon The N1s spectrum reveals four kinds of N in the synthetic BNGS-2-900 sample as shown in Figure 3d, including pyridinic N (398.2 eV, ca. 2.92 at.%), pyrrolic N (399.5 eV, ca. 1.16 at.%) and graphitic N (401.2 eV, ca. 0.76 at.%)^45^.

In order to illustrate the effect of iron species on the formation of BNGS, the sample synthesized by carbonizing the precursor that only introducing [Fe(CN)_6_]^3−^ ions into NR resins (the product named as NGS) and the sample derived from the carbonization of NR resins (the product named as NC) were also prepared, respectively. The XPS spectra were shown in Figure S1 and the corresponding analyzed results were displayed in Table S1. It can be seen that the nitrogen content in NGS is higher than that of the NC, indicating the iron specie have the effect for saving N during the synthesis of BNGS. Besides, the B, N-codoped carbon materials (denoted as BNC) synthesized by the same route only without using [Fe(CN)_6_]^3−^ was also studied. As the XPS spectra shown in Figure S2 and Table 1, there were many B-N and N-B bonds exhibited in the BNC sample. When introducing iron species into NR, there was almost no B-N bond observed (BNGS-2-900 sample). It is further demonstrated that the iron species also play the role of preventing the direct combination between B and N, namely it could avoid the formation of covalent BN. Based on the above analyses, it can be concluded that the iron species not only play as the graphitization catalyst but also play as the role of saving effect for N in BNGS, more important, the iron species also play the role of avoiding formation of covalent BN during the synthesis of BNGS. Additionally, the BNGS synthesized from different concentrate of HBO_3_ and with different carbonized temperature were also studied. It was demonstrated that the contents of B and N in the synthetic BNGS samples could be controllable by tuning the experimental parameters (see Figure S3, S4 and Table S1).

**Figure 4:**
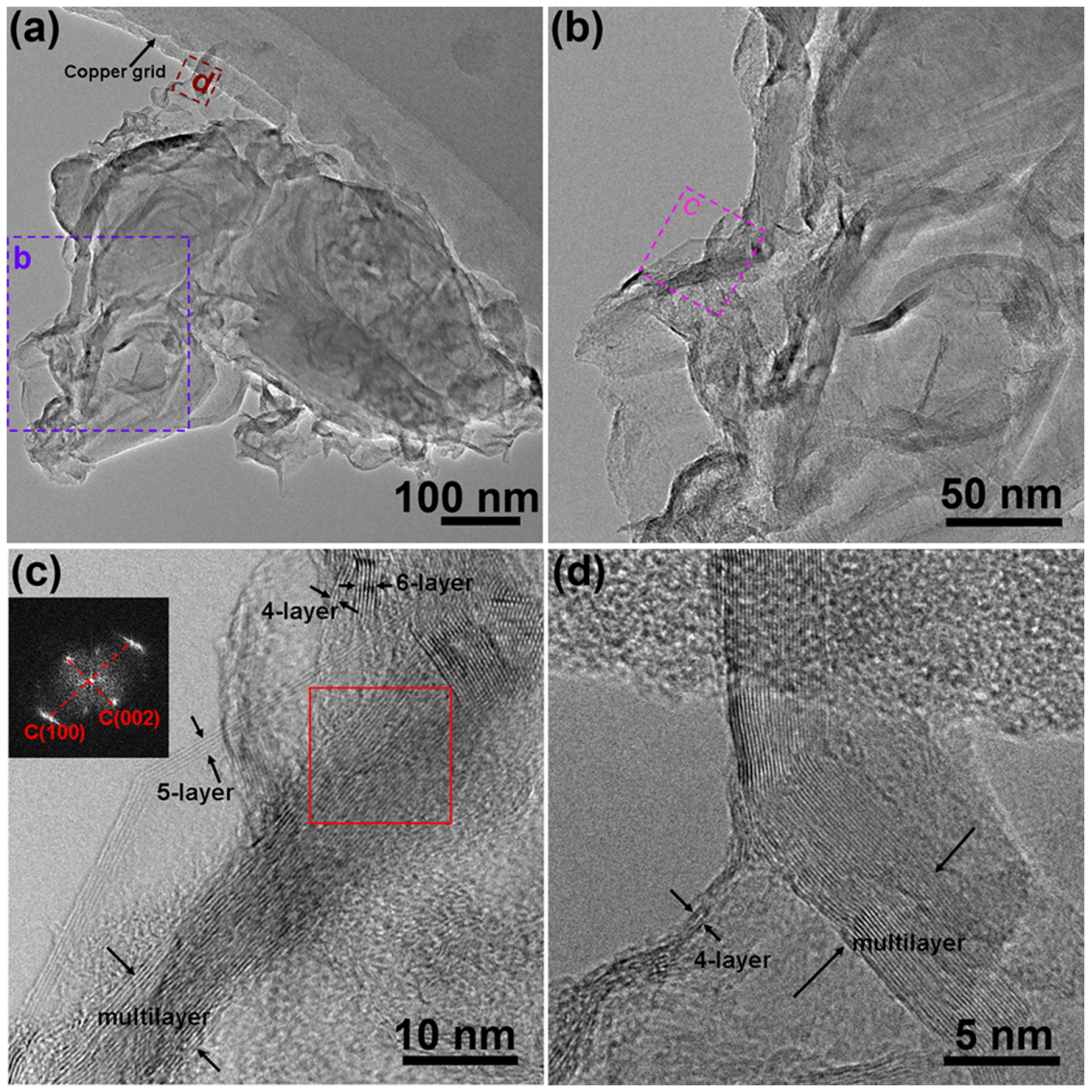
(a) The TEM images of the synthetic BNGS-2-900. (b) is the enlarged image of (a); (c) and (d) are the high-resolution images of (b) and (a), respectively; The Fourier transform (FT) image in the inset of (c) tested on the selected area of red box.

Transmission electron microscopy (TEM) was used to investigate the microscopic structure of BNGS-2-900. As displayed in Figure 4a and Figure 4b, the BNGS-2-900 shows aggregated nanosheets morphology. The HRTEM images further indicate the BNGS-2-900 is composed of four-layer, five-layer and multi-layer graphitic nanosheets (Figure 4c and 4d). Fourier transform (FT) image in Figure 3c displays the (100) and (002) planes of graphitic carbon. The N_2_ adsorption–desorption measurement and the corresponding pore size distribution were shown in Figure S5. The BET specific surface area of the BNGS-2-900 is 548.7 m^2^ g^−1^ and most of the pore are distributed in the range of 5 ~ 10 nm, which is favourable for the ORR owing to the mesopore nanostructures could provide large amounts of potential active sites and channels for reactant/product transfer^45^,^46^.

**Figure 5:**
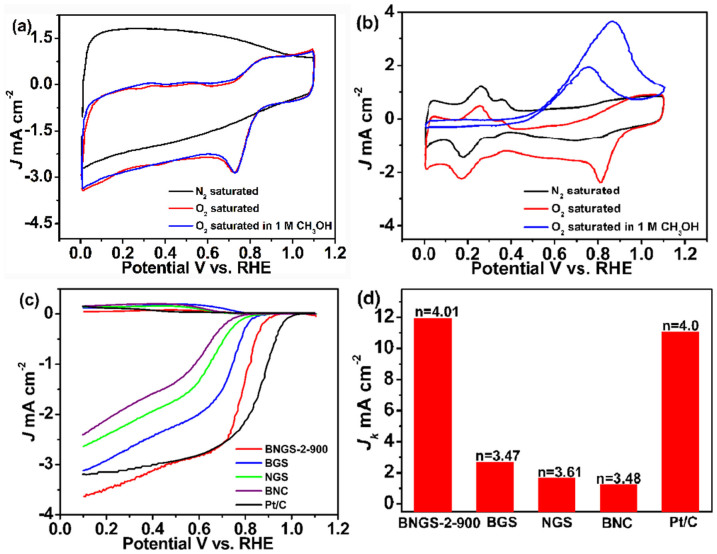
CV curves for ORR on (a) BNGS-2-900 and (b) Pt/C electrodes in N_2_- and O_2_-saturated 0.1 M KOH electrolyte with and without 1.0 M CH_3_OH at a scan rate of 50 mV s^−1^. (c) RRDE voltammetric response in O_2_-saturated 0.1 M KOH electrolyte at a scan rate of 5 mV s^−1^ and (d) electrochemical activity given as the kinetic current density (*J*_K_) at 0.7 V for Pt/C, BNGS-2-900, and all compared electrodes; The electrode rotation rate was 1600 rpm, and the Pt ring electrode was polarized at 1.2 V.

The electrocatalytic performance of BNGS for the ORR was first evaluated using a three-electrode system in a 0.1 M KOH solution saturated with N_2_ or O_2_ by cyclic voltammetry (CV) with a scan rate of 50 mV s^−1^. In the N_2_-saturated electrolyte, featureless voltammetric currents within the potential range of 0.1 ~ 1.1 V were observed for BNGS-2-900 as shown in Figure 5a, only the non-Faradic current characteristic of double-layer charge–discharge appears. In contrast, when the electrolyte was saturated with O_2_, a cathodic peak centered at 0.87 V could be detected, indicating pronounced electrocatalytic activity towards ORR of BNGS-2-900. Additionally, the crossover effect of BNGS-2-900 and commercial Pt/C catalyst (20 wt.% Pt on Vulcan XC-72R) against the electrooxidation of methanol in the presence of 1.0 M CH_3_OH was also performed. As can be seen from Figure 5b, the Pt/C shows a pair of peaks at 0.75 V and 0.86 V, which are attributed to the peaks of methanol electrooxidation, meanwhile the cathodic peak for the ORR disappears. In contrast, there is no obvious change in the CV curve on BNGS-2-900 under the same experimental conditions (Figure 5a). It is demonstrated that BNGS-2-900 electrode has high selectivity and good stability for the ORR with respect to Pt/C. The excellent methanol tolerant property will make it as a promising methanol tolerant cathode catalyst for direct methanol fuel cells (DMFCs).

Linear sweep voltammetry (LSV) on a rotating ring disk electrode (RRDE) was obtained to further investigate the ORR activity of BNGS. The corresponding data about NGS, BNC and Pt/C catalysts were also provided for comparison. Furthermore, the B-doped graphene nanosheets (BGS) synthesized from graphite oxide was also studied (the detailed synthesis was displayed in experiment section). The loadings on the electrodes for all the catalysts are in the range of 200 ~ 205 μg cm^−2^ by weighting method as shown in Table S2.The onset potential (*E*_onset_) of ORR is an important criterion to evaluate the activity of an electrocatalyst. As shown in Figure 5c, the *E*_onset_ of all doped-carbon catalysts are negatively shifted related to the *E*_onset_ value measured for the Pt/C catalyst. Notably, the *E*_onset_ value for ORR on the BNGS-2-900 electrode was about 0.95 V (vs. RHE) versus 1.01 V (vs. RHE) on the Pt/C electrode at the current cut-off of −0.0052 mA cm^−2^ which is only 51 mV difference. The *E*_onset_ of the BNGS-2-900 is much higher than that of the BGS, NGS and BNC electrodes, implying the synergistic effect between B and N, and the rapid electrons transport of graphitic nanosheets in BNGS-2-900 would be favourable for the ORR performance. Moreover, combining the above XPS analyses, the B atoms mainly attach to C atoms in the network of graphitic carbon, meanwhile the N atoms exist in the formation of pyridinic, pyrrolic and graphitic N possess has lone-pair electrons^47^. The characterizations make the synthetic BNGS-2-900 exhibit good performance in ORR. The *E*_onset_ of the BNGS-2-900 is also much higher than that of the reported B,N-codoped graphene and nanocarbon^38^,^48^,^49^. Besides, the ORR current increases sharply for BNGS-2-900 when the potential moves from 1.1 to 0.1 V. Both the BNGS-2-900 and Pt/C catalysts exhibit the same current density at the potential of 0.65 V.

The good ORR performance requires the catalyst has the followed characteristics^50^. Firstly, a high turnover frequency, namely the number of catalytic reactions that can occur at a specific in a second is large, would directly affect the current of ORR. Secondly, a high electronic conductivity of the catalyst support is favorable for the transform of conduct electrons to/from the catalytic site. Finally, the electron transfer number “*n*” in the oxygen reduction process is much more close to four, indicating a better performance of the catalyst towards ORR. Generally, oxygen reduction in an alkaline medium proceeds either reduced to peroxide species (HO_2_^−^) as an intermediate through a two-electron transfer process and then further reduction to OH^−^, or it can be directly reduced to OH^−^ through a four-electron transfer pathway^51^,^52^. Of course, the latter is a ideal and efficient energy conversion process. To get insight into the ORR mechanism of the different catalysts, the ORR pathways catalyzed by all the compared catalysts were also evaluated by RRDE measurements of Figure 4c, and the results are shown in Figure 5d. The electron transfer number (*n*) was determined by the followed equations: 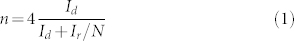
where *I*_r_ is ring current, *I*_d_ is disk current, and *N* is current collection efficiency of the Pt ring (N = 0.37). The electron transfer number n in the ORR of the BNGS-2-900, BGS, NGS, BNC and Pt/C electrodes were calculated to be 4.01, 3.47, 3.61, 3.48 and 4.0, respectively. This suggests that BNGS-2-900 shows a more efficient quasi-four-electron process with water as the product as that of the Pt/C catalyst. As compared, the bare RRDE electrode was also tested in O_2_-saturated 0.1 M KOH electrolyte as shown in Figure S6. The bare RRDE electrode exhibited a two-electron process for ORR, implying the support has no contribution for the directly reducing O_2_ to water of BNGS-2-900 catalyst.

The specific kinetic current densities (*J*_k_) associated with the intrinsic activity of the catalysts can be obtained by Koutecky-Levich equation: 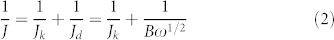
where *J*_k_ is the kinetic current density, *J*_d_ is the diffusion-limited current density, *ω* is the angular frequency of rotation. The *B* parameter is defined as 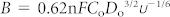, where *n* is the overall number of electrons, F is the Faraday constant (96485 C mol^−1^), *C*_o_ is the concentration of molecular oxygen in the electrolyte (1.2 × 10^−6^ mol cm^−3^), *D*_o_ is the diffusion coefficient of the molecular O_2_ in 0.1 M KOH solution (1.9 × 10^−5^ cm^2^ s^−1^), and 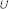 is the viscosity of the electrolyte (0.01 cm^2^ s^−1^)^53^. The *J*_k_ values were about 11.9, 2.7, 1.7, 1.3 and 11.1 mA cm^−2^ for BNGS-2-900, BGS, NGS, BNC and Pt/C evaluated from the disk current at 0.7 V, respectively. The higher current density and the four-electron process indicate the BNGS-2-900 is a kind of high efficient ORR catalyst. The electronic conductivity test results were shown in Table S3. It can be seen that the BNGS-2-900 exhibits the largest conductivity compared with that of the BGS, NGS and BNC catalysts, suggesting the BNGS-2-900 was more favorable for the electron transfer. Additionally, the LSV curves for BNGS-2-900 with different loading on electrodes were also studied (Figure S7). It can be observed an increase in the absolute value of the limiting current and the onset potential has not obvious change. Notably, the loading of 202.4 μg cm^−2^ exhibited the highest kinetic current density and also much more closer to the four-electron process. The corresponding activity calculated based on the real surface area of BNGS is about 0.01 mA cm^−2^. Due to the BNGS has a large BET surface area, so the specific surface ares activity is lower than the reported catalyst^54^.

Moreover, the corresponding RRDE curves of BNGS synthesized from different carbonized temperature and different concentration of HBO_3_. As shown in Figure S8a, the ORR *E*_onset_ of BNGS-2-800, BNGS-2-1000, BNGS-1-900 and BNGS-3-900 are about 0.87, 0.84, 0.83 and 0.85 V, respectively. The electron number n values are about 4.10, 3.98, 3.96 and 3.87 for BNGS-2-800, BNGS-2-1000, BNGS-1-900 and BNGS-3-900, respectively, the *J*_k_ values are about 8.62, 7.95, 7.64 and 8.26 (Figure S8b). It can be concluded that BNGS-2-900 exhibited the best performance towards ORR due to the appropriate crystallinity and the moderate content of B and N.

**Figure 6:**
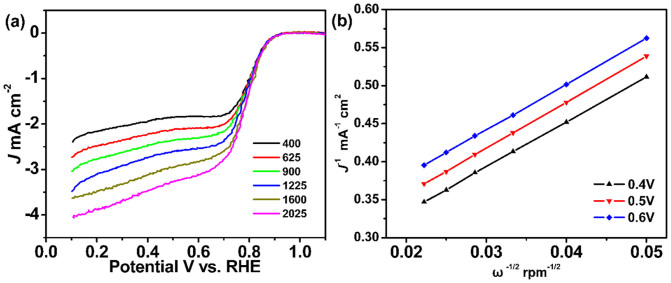
(a) LSV curves for BNGS-2-900 on a RDE in an O_2_-saturated 0.1 M KOH solution with various rotating speeds at scan rates of 5 mV s^−1^. (b) Koutecky−Levich plots of J^−1^ vs. ω^−1/2^ at different potential for BNGS-2-900 obtained from the LSV curves in (a).

Figure 6a shows the LSV curves for BNGS-2-900 on rotating disk electrode (RDE) at various rotating speeds. It could be observed that the limiting current density increases with the increasing of rotating speeds. The ORR kinetics of the BNGS-2-900 electrode was further analyzed by the Koutecky–Levich plots of J^−1^ vs. ω^−1/2^ calculated based on the LSV curves in Figure 6a. As shown in Figure 6b, the plots exhibit good linear at the potential of 0.4, 0.5 and 0.6 V, demonstrating the first-order kinetics of BNGS-2-900 for ORR. Moreover, the electron transfer number n of BNGS-2-900 towards ORR is about 3.99 calculated based on the Koutecky–Levich equation, which is consistence with the above RRDE results.

**Figure 7:**
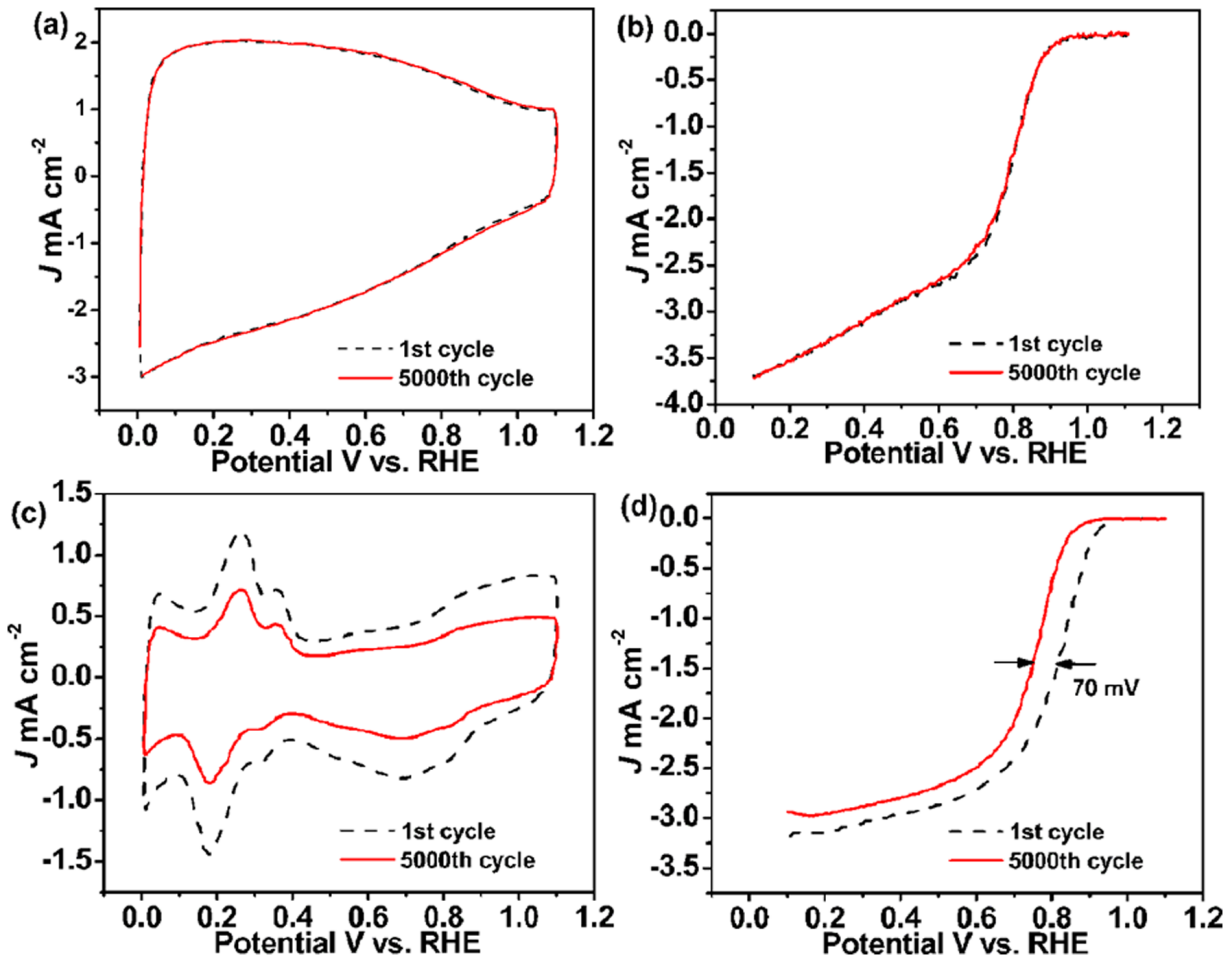
Stability tests of the catalysts: The CVs before and after 5000 cycles on (a) BNGS-2-900 and (c) Pt/C electrodes in an N_2_-saturated 0.1 M L^−1^ KOH electrolyte at a scan rate of 50 mV s^−1^; The ORR curves before and after 5000 cycles on the (b) BNGS-2-900 and (d) Pt/C electrodes at 1600 rpm in O_2_-saturated 0.1 M L^−1^ KOH solution at a scan rate: 5 mV s^−1^.

The stability of catalyst towards ORR is as important as the activity for any electrocatalyst to be considered for realistic applications base on the U.S. Department of Energy's accelerated durability test. To evaluate the stability of the BNGS-2-900 catalyst, the cyclic CVs curves tested in N_2_-stratured 0.1 M KOH electrolyte and the ORR polarization curves in O_2_-saturated 0.1 KOH before and after 5000 cycles were carried out, and the corresponding data for Pt/C were also provided for comparison. As the CVs shown in Figure 7a, there was almost no change on BNGS-2-900 after 5000 cycles, while the corresponding CVs for Pt/C decreased obviously (Figure 7c), indicating the electrochemical active surface area (EASA) for Pt/C decreased sharply after cycling test. This is direct evidence of the better stability of BNGS-2-900 compared with Pt/C catalyst. More importantly, the ORR polarization curve of BNGS-9-200 still has the same *E*_onset_ as the initial value of 0.95 V vs. RHE (Figure 7b). However, although the ORR initial *E*_onset_ of Pt/C catalyst is positive 51 mV compared to BNGS-2-900 at the beginning (Figure 7d), the half-wave potential on negatively shift 70 mV after 5000 cycles, so the final *E*_onset_ for Pt/C is lower than that of BNGS-2-900. The improved stability might originate from the graphitic nanosheets in BNGS-2-900 is more stable than the Vulcan XC-72 in Pt/C catalyst. The BNGS-2-900 catalyst after 5000 cycles was also analyzed by XPS. There is no new peaks could be observed as shown in Figure S9. It is demonstrated that the synthetic doped carbon catalyst exhibits excellent stability under cycling, because the crystalline carbon in the synthetic catalyst possesses good chemical stability. However, the C-O content in the catalyst is about 9.35% calculating based on the total carbon atoms, which is clearly higher than that of the original BNGS-2-900 catalyst of 4.26%. Relatively, the contents of C-N and C-B bonds exhibited almost no change after cycling, indicating that the N and B in the catalyst is hard to be oxidized. Although the C-O groups have some activity towards ORR, compared with the high active groups of C-B and C-N, the C-O groups play little role for ORR in the synthetic BNGS catalyst. Therefore, the synthetic doped carbon catalyst exhibited well electrochemical stability. Also, the synthetic BNGS-2-900 catalyst exhibited good stability after 5000 cycles in 0.1 M KOH + 1.0 M CH_3_OH electrolyte as shown in Figure S10. Therefore, BNGS-2-900 could be used for potential ORR electrocatalyst in the future.

In summary, we have developed a facile and economic method for synthesis of BNGS as an effective catalyst towards ORR. During the synthesis, the iron species not only play as the graphitization catalyst but also play as the saving effect for N. More importantly, the iron species could prevent the direct combination between B and N atoms. Additionally, the N atoms fixed on the backbone of NR also avoided the direct contact between B and N during the synthesis, so the covalent BN would not be existed in the final sample. Moreover, the content of B and N in the synthetic BNGS could be controllable by tuning the concentration of HBO_3_ in the initial ion-exchanged solution and the carbonized terperature. The synthetic BNGS-2-900 exhibits high ORR activity, good selectivity and excellent stability compared to commercial Pt/C catalyst. Moreover, the BNGS-2-900 has much better ORR performance than BGS, NGS and BNC. The superior performance of the BNGS-2-900 for the ORR can be ascribed to the synergistic effect between B and N, and well electronic conductivity of graphitic carbon. This study opens up a new avenue for synthesis of metal-free ORR catalysts with both excellent electrocatalytic activity, selectivity and stability, which is important to the development of new energy conversion devices, such as fuel cells and metal−air batteries.

## Methods

### Synthesis of BNGS

BNGS materials were prepared via a general ion-exchanged process. Briefly, 3.00 mmol K_3_[Fe(CN)_6_] was dissolved in 80 mL deionized (DI) water, then 14.53 g of nitrogen-containing epoxy weak-alkaline anion-exchanged resins (NR) were added. After stirring for 3 h to form NR-[Fe(CN)_6_]^3−^ composite, then 2.00 mmol HBO_3_ was added into the above solution and the suspension was continued stirred for 5 h. After filtration, the prepared NR-[Fe(CN)_6_]^3−^-BO_3_^−^ precursor was dried in an oven at 80°C. Subsequently, the precursor was pyrolyzed at 900°C for 1 h under 80 cm^3^ min^−1^ of argon atmosphere. The pyrolyzed composite was stirred in 200 mL of 4.00 M HCl in order to dissolve the Fe particles in the carbon. Finally, the catalysts were filtered, washed with DI water, and dried in an oven, then the B,N-doped graphitic nanosheets (BNGS) was obtained. The resultant samples were denoted as BNGS-*c*-*T*, where *c* standed for the amount of HBO_3_ for synthesis of BNGS (mmol), *T* standed for the heat treated temperature, so the product was denoted as BNGS-2-900. The N- and B-doping level strongly depends on the concentration of HBO_3_ and the pyrolyzed temperature, so the samples of BNGS-1-900, BNGS-3-900, BNGS-2-800 and BNGS-2-1000 were also prepared, respectively.

### Synthesis of NGS, NC and BNC

For comparison the ORR performance of BNGS with the nitrogen-doped graphitic nanosheets (NGS), the synthetic process was conducted in the absence of HBO_3_ to obtain NGS. The N-doped carbon materials (NC) and B, N-codoped carbon materials (BNC) without graphitization were also synthesized by carbonization of NR and NR-BO^3−^ at 900°C, respectively.

### Synthesis of BGS

Due to N atoms exsit in the NR, so the only B-doped graphitic nanosheets (BGS) could not be prepared by the present method from NR resins. In the contrast experiment, the BGS was synthesized derived from the graphite oxide solution containing HBO_3_ after hydrothermal at 180°C for 12 h for explaining the synergistic effect between B and N for ORR.

All the compared sample names and the corresponding experimental conditions were displayed in Table S4.

### Material characterizations

X-ray diffraction (XRD) patterns were carried on a Bruker D8 Advance diffractometer equipped with Cu K_α_ (λ = 1.5406 Å) radiation and a LynxEye Detector. X-ray photoemission spectroscopy (XPS) analyses were performed on a Kratos-AXIS UL TRA DLD with Al K_α_ radiation source. Raman spectra were tested by a Jobin Yvon HR 800 micro-Raman spectrometer at 457.9 nm. Transmission electron microscopy (TEM) characterization was studied on a JEM-2100 electron microscope (JEOL) with an acceleration voltage of 200 kV. The nitrogen adsorption-desorption isotherms of were performed by using a Micromeritics TriStar II.

### Electrochemical measurements

The electrochemical measurements were performed by using a Pine Instrument biopotentiostat a standard three-electrode system with a platinum foil (1.0 cm^2^) as the counter electrode and a home-made reversible hydrogen electrode (RHE) as the reference electrode. All ORR activities were measured by means of a modulated speed rotator (MSR) rotating ring-disk electrode (RRDE) with a Pt ring (inner/outer-ring diameter: 6.25/7.92 mm) and a glassy carbon disk (diameter: 5.61 mm) at 25°C. The working electrode was prepared as follows: A mixture of the 10 mg electrocatalyst was mixed with 1.8 mL ethanol and 0.2 mL 0.5 wt.% Nafion suspension and ultrasonicated for 1 h to obtain a well-dispersed electrocatalyst “ink”. Then, 10 μL of the electrocatalyst as-prepared ink was transfered onto the surface of the glassy carbon disk of the RRDE and a electrocatalyst thin-layer could be obtained after drying. The ORR activities of the electrocatalysts were tested in O_2_-saturated 0.1 mol L^−1^ KOH aqueous solution in the potential range of 0 ~ 1.1 V (vs. RHE) with a scan rate of 5 mV s^−1^. The ring potential was held at 1.2 V versus RHE.

## Author Contributions

H.F. directed the research. L.W., P.Y., L.Z., D.Z. & J.Y. performed the experiments and characterizations. L.W. wrote the manuscript. C.T. contributed to TEM analysis. Z.W. contributed to XPS analysis. R.W. contributed to the electrochemical analysis.

## Supplementary Material

Supplementary Information supporting information

## Figures and Tables

**Table 1:** The high-resolution XPS spectrum analytic results of B1s and N1s for BNGS-2-900 and BNC samples

	B atoms		N atoms	
samples	Bond types	Content (at.%)	Bond types	Content (at.%)
BNGS-2-900	B-C	3.87	Pyridinic N	3.33
	B-O	0.53	Pyrrolic N	1.26
			Graphitic N	0.53
BNC	B-N	1.16	N-B	1.06
	B-C	0.05	Pyridinic N	0.96
	B-O	1.54	Pyrrolic N	1.19
