# Supplementary material for: B and N isolate-doped graphitic carbon nanosheets from nitrogen-containing ion-exchanged resins for enhanced oxygen reduction
Source: Sci Rep. 2014 Jun 5;4:5184. doi: 10.1038/srep05184 (PMC4046170; doi:10.1038/srep05184)
Supplement: Supplementary Information — supporting information [file srep05184-s1.doc]

Supplementary information

B and N isolate-doped graphitic carbon nanosheets from nitrogen-containing ion-exchanged resins for enhanced oxygen reduction

Lei Wang, Peng Yu, Lu Zhao, Chungui Tian, Dongdong Zhao, Wei Zhou, Jie Yin, Ruihong Wang & Honggang Fu*


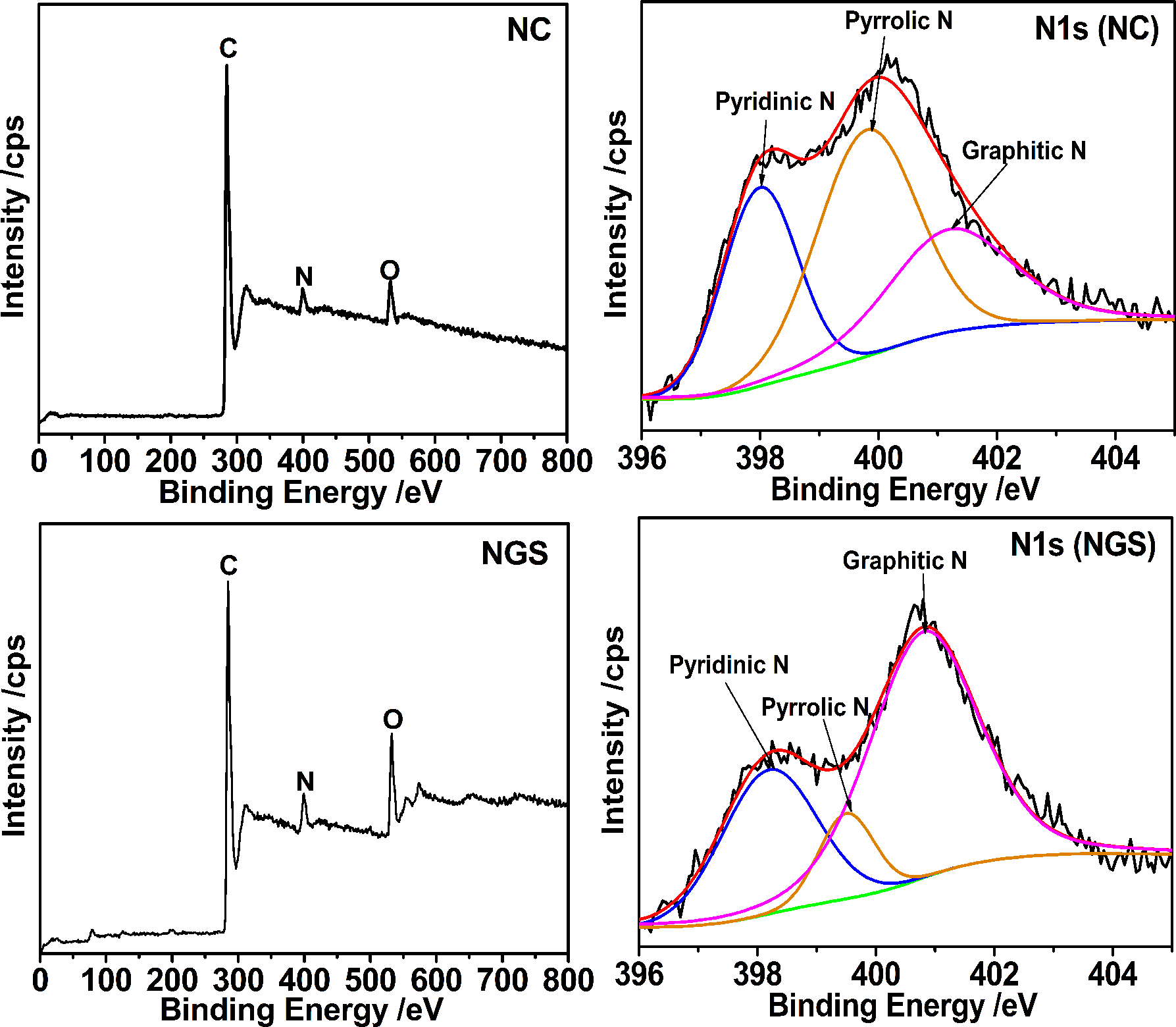


**Figure S1** Survey spectrum and high-resolution XPS spectrum of NC and NGS samples.

**
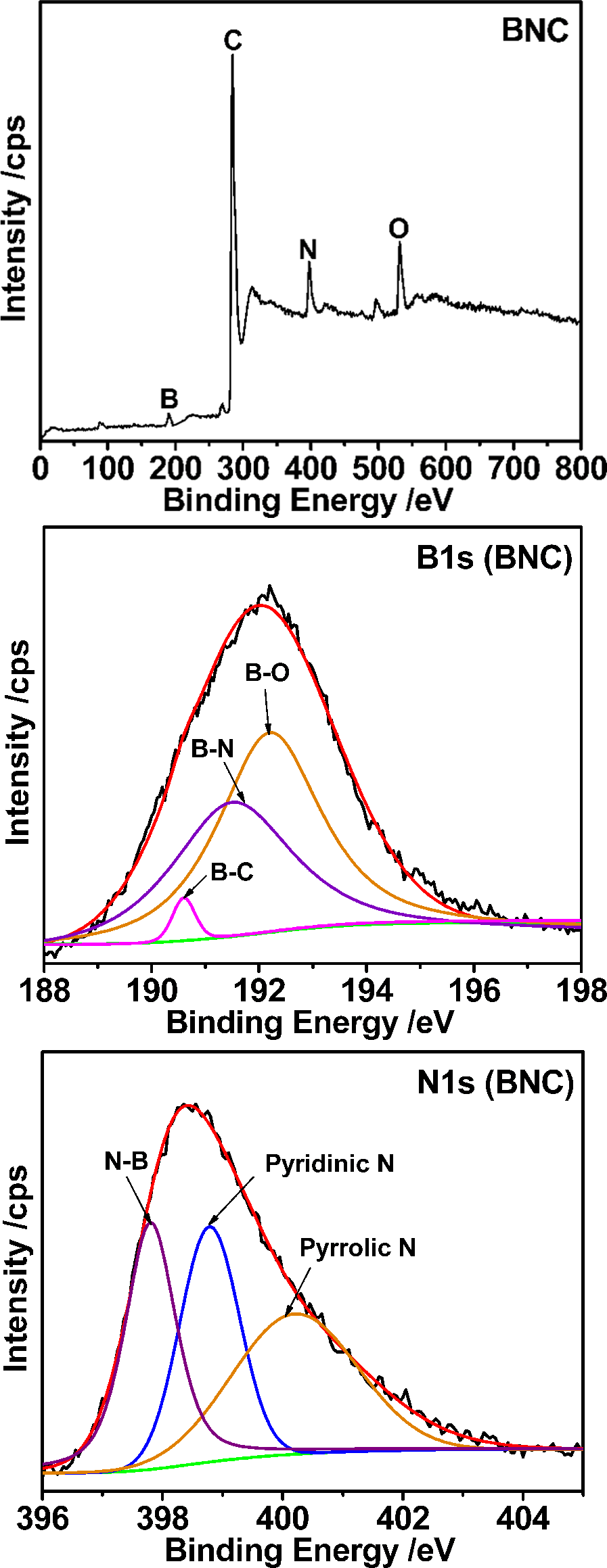
**

**Figure S2** Survey spectrum and high-resolution XPS spectrum of BNC sample.

**
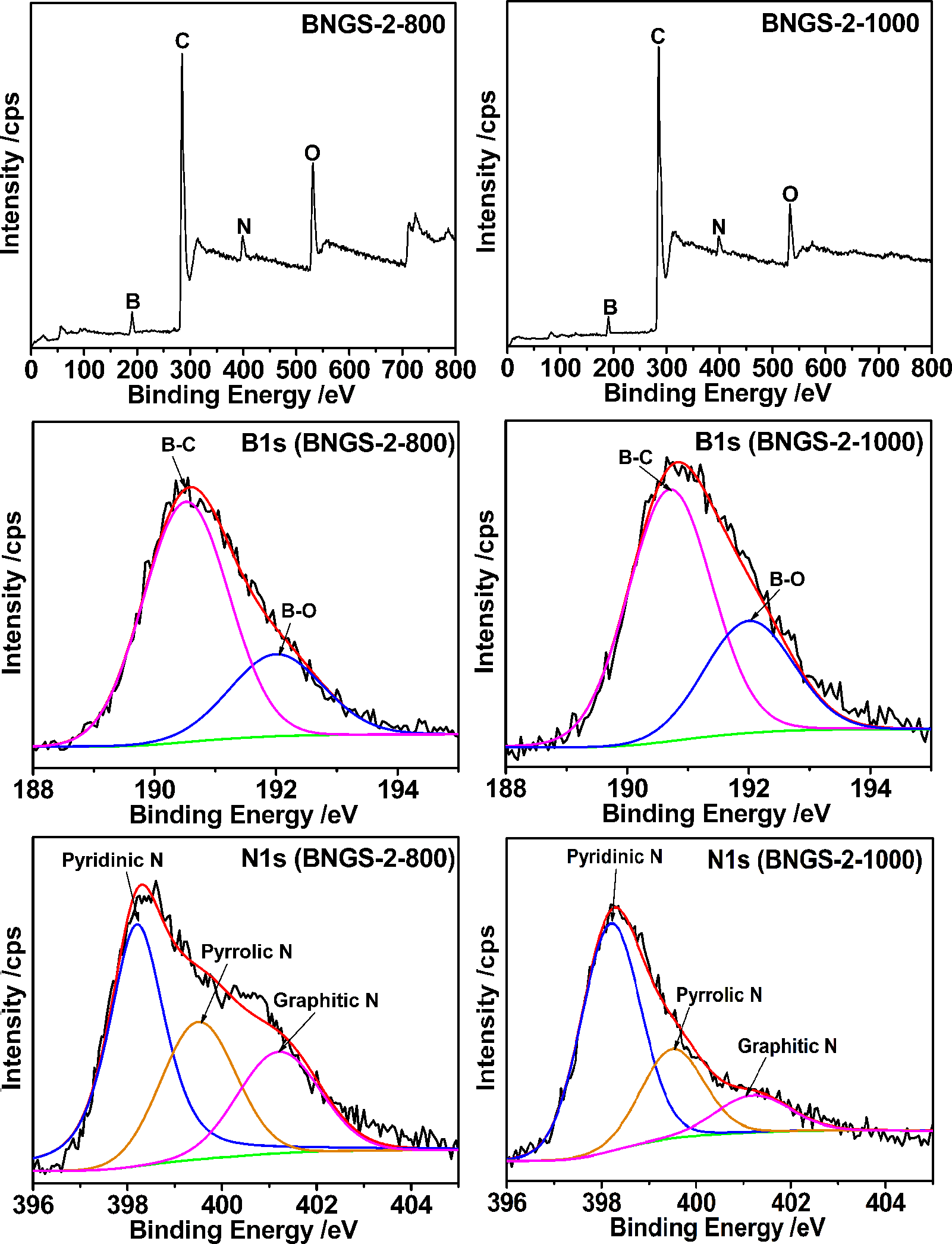
**

**Figure S3** XPS spectra and high-resolution XPS spectra of BNGS-2-800 and BNGS-2-1000 samples.

**
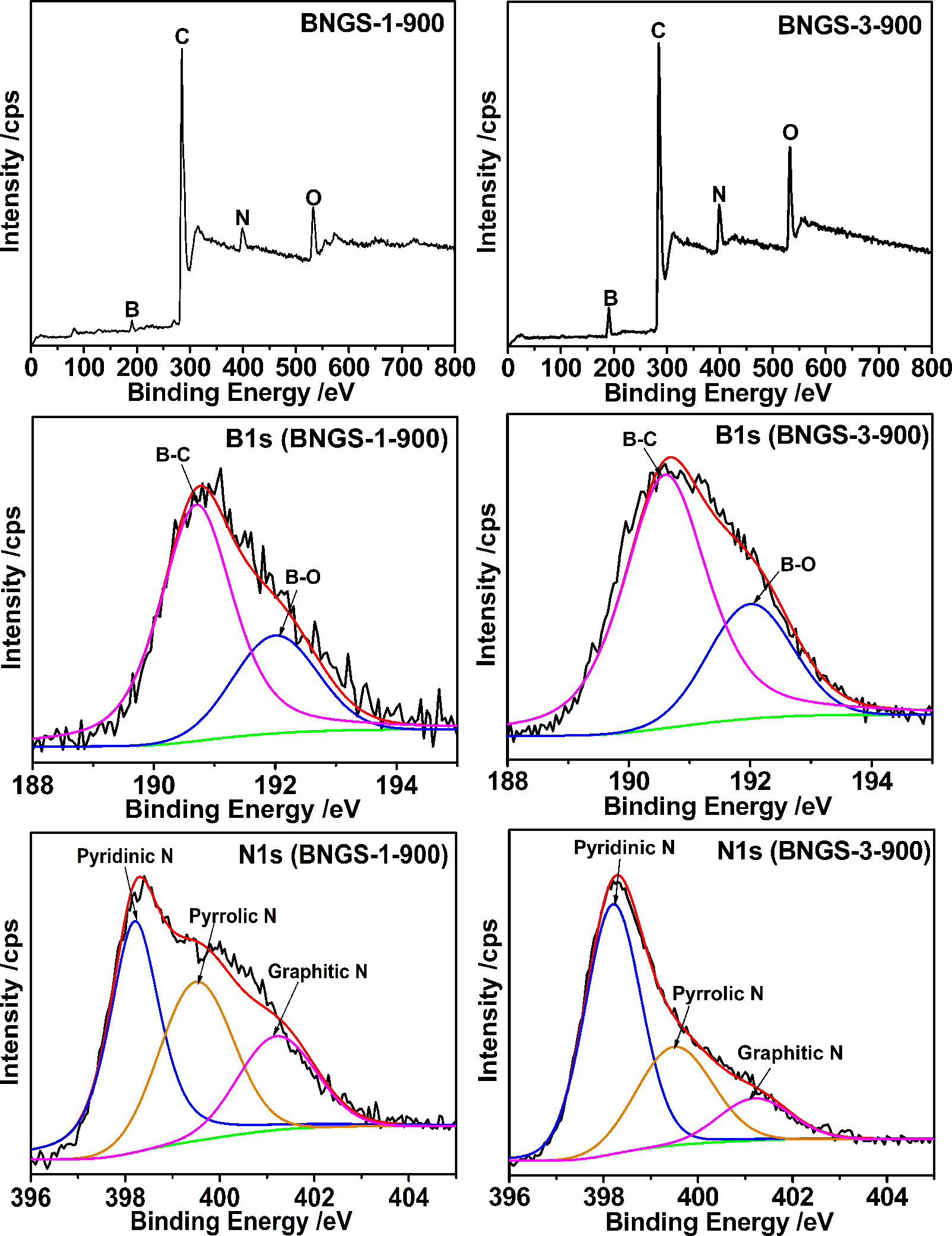
**

**Figure S4** XPS spectra and high-resolution XPS spectra of BNGS-1-900 and BNGS-3-900 samples.

**Table S1 XPS analyzed results of the synthetic samples.**

| samples | B content (At.%) | | | N content (At.%) | | | |
| --- | --- | --- | --- | --- | --- | --- | --- |
|  | Total | B-C | B-O | Total | Pyridinic N | Pyrrolic N | Graphitic N |
| BNGS-1-900 | 2.56 | 1.85 | 0.71 | 5.42 | 2.37 | 1.88 | 1.17 |
| BNGS-2-800 | 4.75 | 3.45 | 1.30 | 6.64 | 3.19 | 1.90 | 1.55 |
| BNGS-2-900 | 4.40 | 3.87 | 0.53 | 5.12 | 3.33 | 1.26 | 0.53 |
| BNGS-2-1000 | 3.36 | 2.31 | 1.05 | 4.13 | 2.15 | 1.38 | 0.60 |
| BNGS-3-900 | 5.13 | 3.75 | 1.38 | 5.95 | 3.49 | 1.75 | 0.71 |
| NGS |  |  |  | 3.13 | 0.88 | 0.31 | 1.94 |
| NC |  |  |  | 2.1 | 0.59 | 0.91 | 0.60 |

**
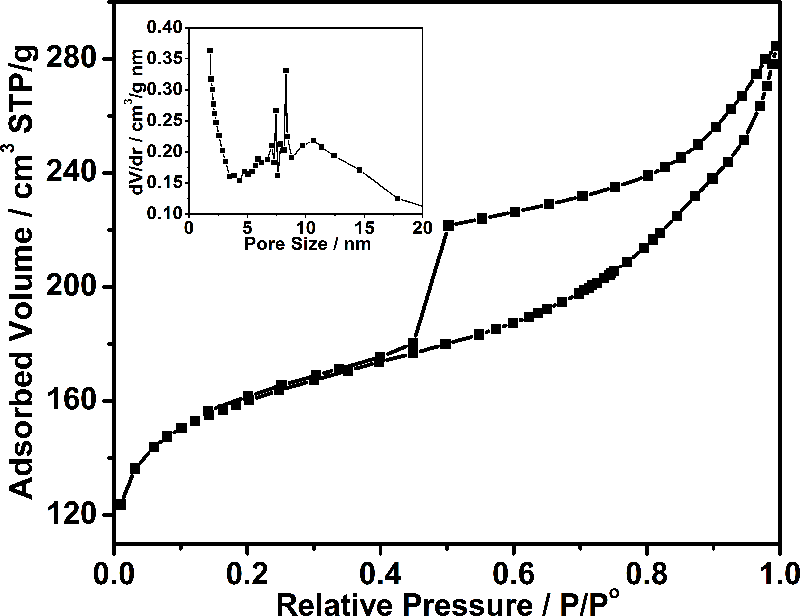
**

**Figure S5** N_2_ adsorption-desorption isotherms of BNGS-2-900 sample, and inset is the corresponding pore size distribution.

**Table S2** The loadings on the electrodes for all the compared catalysts.

| Samples | Loading on electrodes (μg cm^–2^) |
| --- | --- |
| BNGS-2-800 | 203.6 |
| BNGS-2-900 | 202.4 |
| BNGS-2-1000 | 201.8 |
| BNGS-1-900 | 201.3 |
| BNGS-3-900 | 200.9 |
| NGS | 203.7 |
| BNC | 204.4 |
| BGS | 200.2 |
| Pt/C | 202.6 |

**Table S3** The conductivity test results of all the samples.

| Samples | Resistivity ρ (Ω•cm) | Conductivity (S cm^−1^) |
| --- | --- | --- |
| BNGS-2-800 | 0.0296 | 33.78 |
| BNGS-2-900 | 0.0262 | 38.17 |
| BNGS-2-1000 | 0.0221 | 45.25 |
| BNGS-1-900 | 0.0316 | 31.65 |
| BNGS-3-900 | 0.0289 | 34.60 |
| NGS | 0.0374 | 26.74 |
| BNC | 0.0412 | 24.27 |
| NC | 0.0447 | 22.37 |
| BGS | 0.0456 | 21.93 |

**
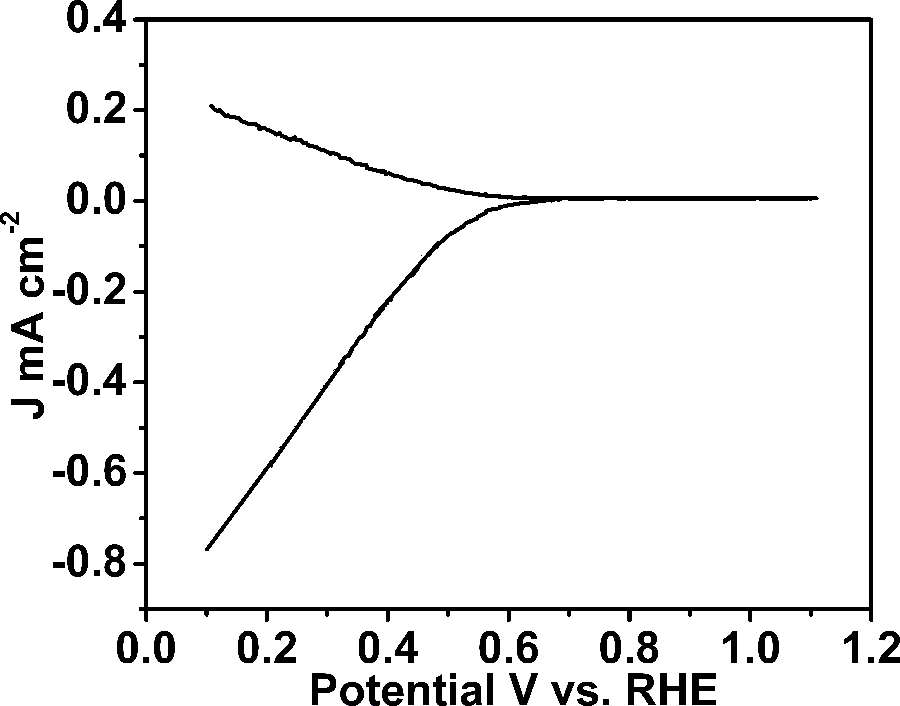
**

**Figure S6.** The RRDE voltammetric response of bare glass carbon electrode in O_2_-saturated 0.1 M KOH electrolyte at a scan rate of 5 mV s^–1^.

**
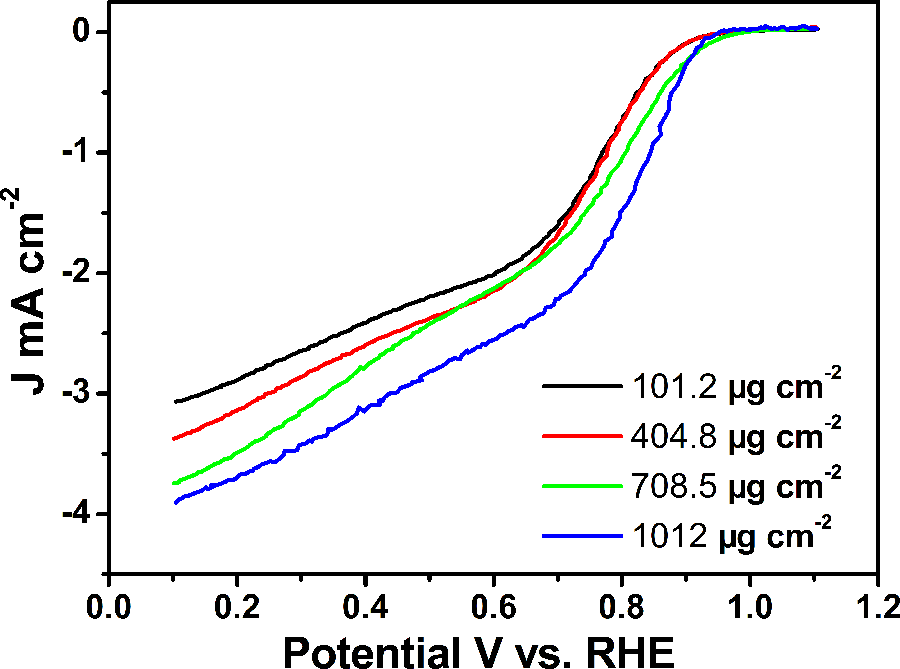
**

**Figure S7.**  LSV curves for BNGS-2-900 with different loading on a RDE in an O_2_-saturated 0.1 M KOH solution.


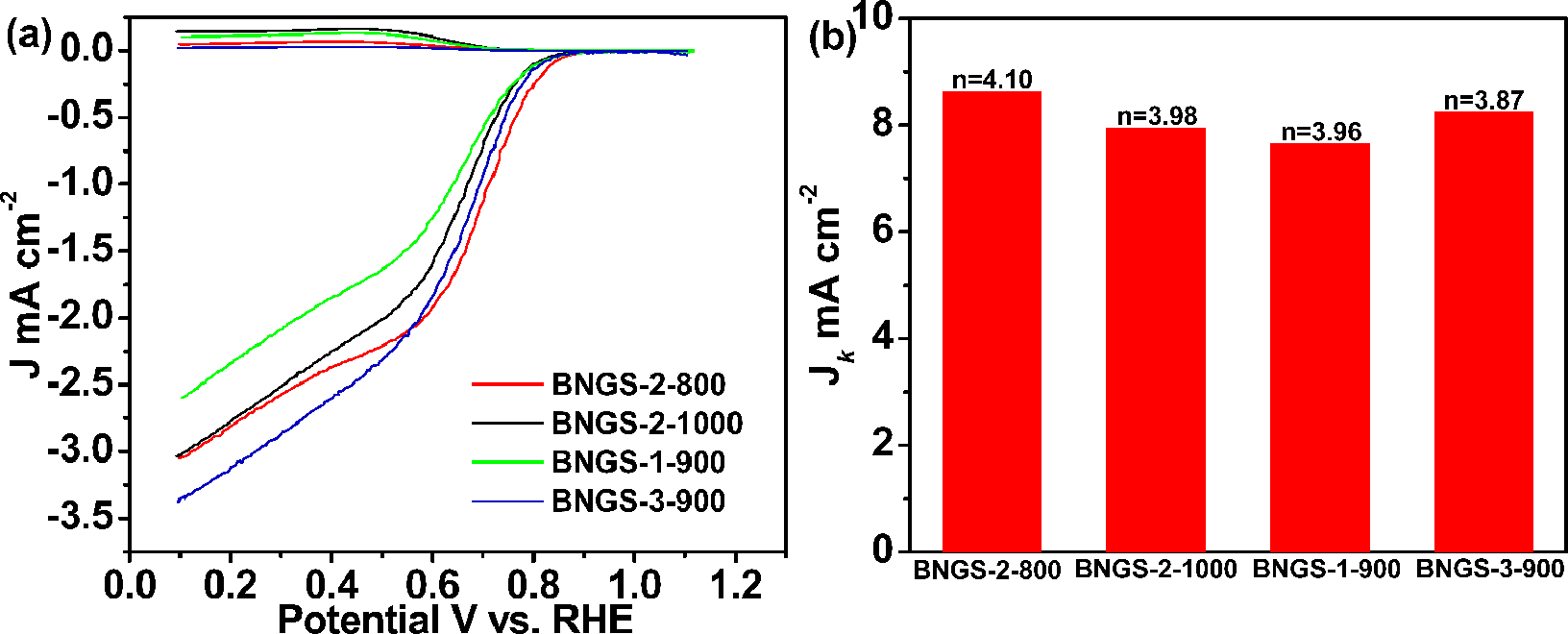


**Figure S8.** (a) RRDE voltammetric response for the ORR in O_2_-saturated 0.1 M KOH at a scan rate of 5 mV s^–1^and (d) electrochemical activity given as the kinetic-limiting current density (*J*_K_) at 0.7 V for all compared BNGS electrodes.

**Table S4 The experimental parameters of the synthesized BNGS.**

| Samples | The usage of HBO_3_ (mmol) | The usage of K_4_[Fe(CN)_6_] (mmol) | Heated temperature (^o^C) |
| --- | --- | --- | --- |
| BNGS-2-800 | 2.00 | 3.00 | 800 |
| BNGS-2-900 | 2.00 | 3.00 | 900 |
| BNGS-2-1000 | 2.00 | 3.00 | 1000 |
| BNGS-1-900 | 1.00 | 3.00 | 900 |
| BNGS-3-900 | 3.00 | 3.00 | 900 |
| NGS |  | 3.00 | 900 |
| BNC | 2.00 |  | 900 |
| NC |  |  | 900 |
| BGS | 30 mL 2 mg mL^–1^ GO solution containing 0.05 mmol HBO_3_ was hydrothermal treated at 180 ^o^C for 12 h | | |


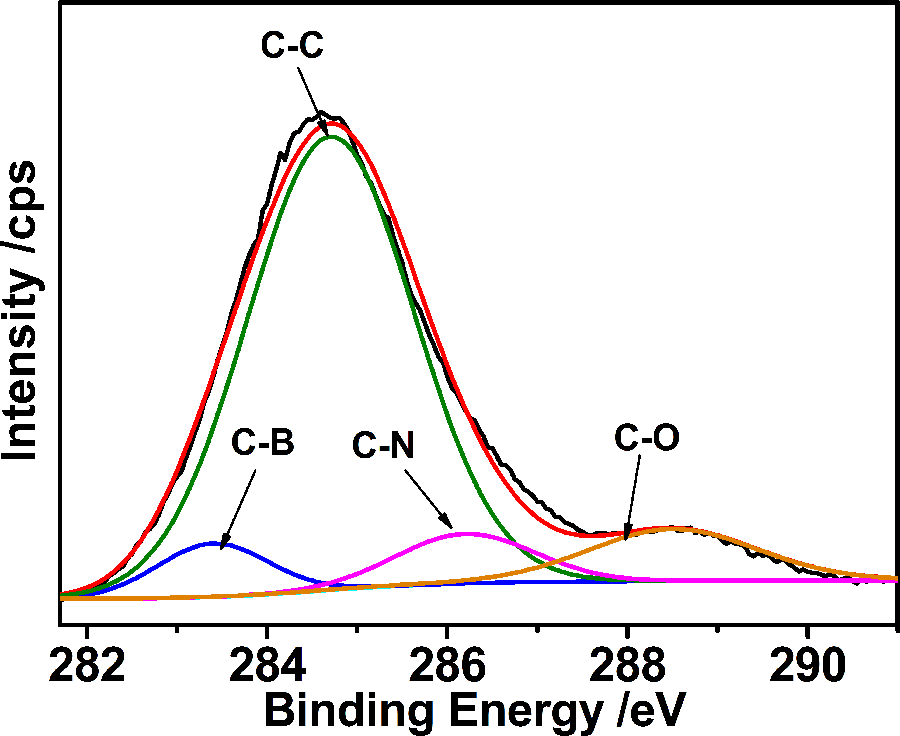


**Figure 9.** C1s of BNGS-2-900 catalyst tested in O_2_-saturated 0.1 M KOH after 5000 cycles.


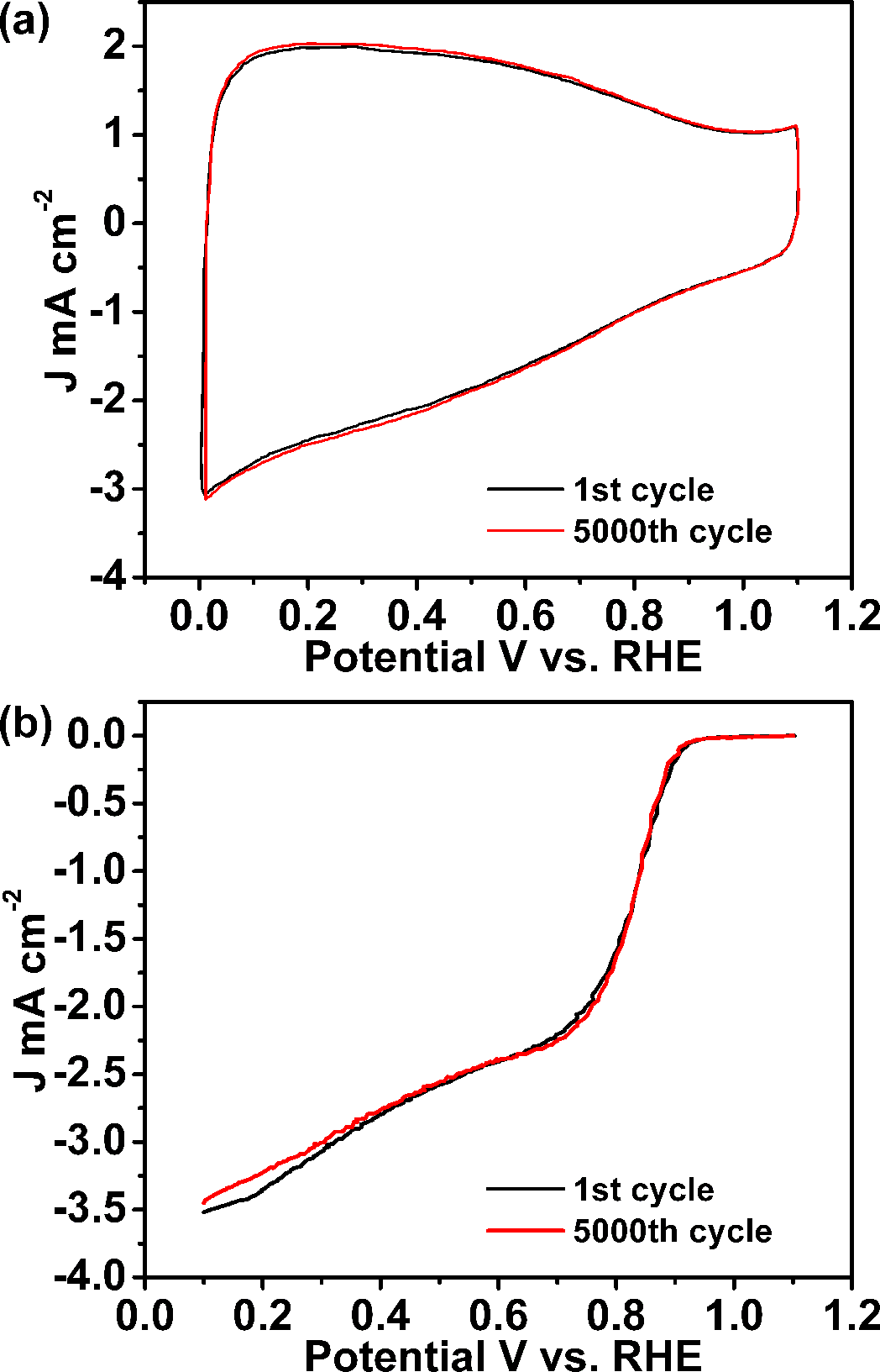


**Figure S10.** Stability tests of the BNGS-2-900 catalyst in 0.1 M L^–1^ KOH+1.0 M CH_3_OH electrolyte: (a) The CVs before and after 5000 cycles on in N_2_-saturated electrolyte at a scan rate of 50 mV s^–1^. (b) The ORR curves before and after 5000 cycles at 1600 rpm in O_2_-saturated 0.1 M L^–1^ KOH solution at a scan rate: 5 mV s^–1^.
